# Supplementary material for: Platelet transcription factors license the pro-inflammatory cytokine response of human monocytes
Source: EMBO Mol Med. 2024 Jul 8;16(8):6. doi: 10.1038/s44321-024-00093-3 (PMC11319489; doi:10.1038/s44321-024-00093-3)
Supplement: Supplementary file 1 — Appendix [file 44321_2024_93_MOESM1_ESM.pdf]

Appendix

Platelet transcription factors license the pro-inflammatory cytokine response of human monocytes.

Ibrahim Hawwari, Lukas Rossnagel, Nathalia Sofia Rosero Reyes, Salie Maasewerd, Marius Jentzsch, Agnieszka Demczuk, Lino L Teichmann, Lisa Meffert, Damien Bertheloot, Lucas S. Ribeiro, Sebastian Kallabis, Felix Meissner, Moshe Arditi, Asli Atici, Magali Noval Rivas, and Bernardo S. Franklin.

Table of Content

Appendix Fig S1 - Staining Controls for the detection of ASC specks in primary human monocytes.---------------------------------------2

Appendix Fig. S2 - Platelet depletion induces transcriptional reprogramming in primary human monocytes. ---------------------------------------4

Appendix Fig. S3 - Bulk RNASeq analysis of FACS-sorted murine monocytes from platelet-depleted mice.---------------------------------------5

Appendix Data S4 - S5 - Platelets regulate cytokine secretion in human monocytes in trans and independently of classical platelet-monocyte crosstalk mechanisms.------------------------------7

Appendix Fig. S4 - Platelets modulate monocyte cytokine responses in trans.---------------------------------------9

Appendix Fig. S5: Platelet effects are independent of classical cross-talk mechanisms and sialic acids.---------------------------------------11

Appendix Fig S6 - Analysis of Mass speck proteomics combined with Stable isotope labeling with amino acids in cell culture (SILAC)---------------------------------------13

Appendix Fig. S7 - The IKK inhibitor (BAY11-7082) prevents platelet activation.---------------------------------------14

Appendix Fig. S8 - Fractionation of platelet releasates and isolation of platelet vesicles.---------------------------------------15

Appendix References---------------------------------------17

Appendix Fig S1 - Staining Controls for the detection of ASC specks in primary human monocytes.

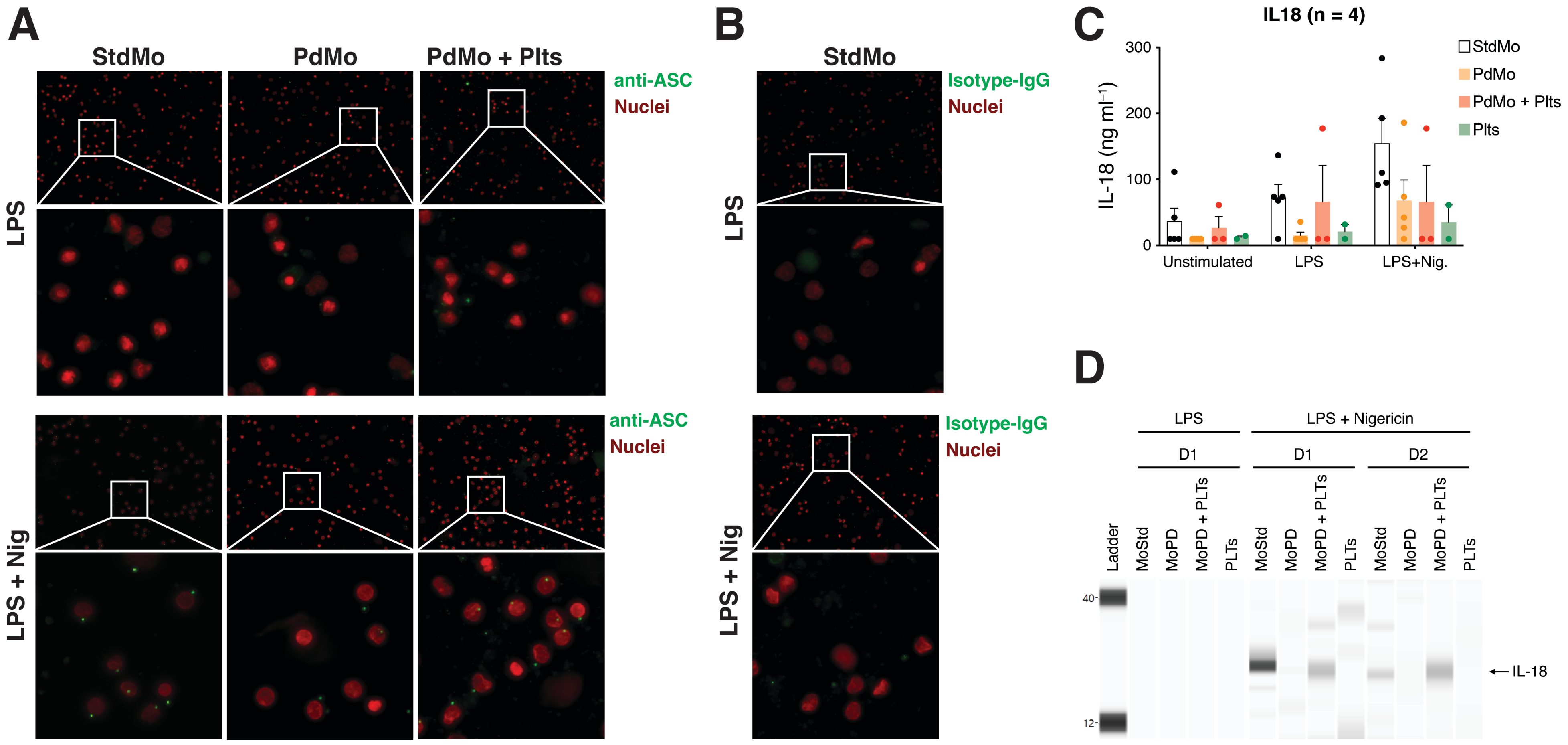

(**A-B**) Representative images of immunostaining and confocal imaging of human ASC speck formation in StDMo, PdMo, or PdMo + Plts that were primed with LPS (2 ng ml−1, 3 h) and left unstimulated (top rows), or stimulated with Nigericin (10 μM, 90 min, bottom rows) in the presence of VX-765 (50 μM) to prevent ASC speck release. Samples were blocked with 1:10 human FcR blocking reagent for 30 minutes at room temperature and stained with directly labeled anti-ASC-488 (A) or the same amount of directly labeled IgG1 control (B). Images are from one representative of 3 independent experiments.

(**C**) IL-18 concentrations measured by Luminex in CFS of LPS-primed StdMo, PdMo, or PdMo + Plts (50 platelets:monocyte). Each symbol represents one independent experiment or blood donor (n = 5 for monocytes, and n = 2 for platelets alone).

(**D**) Maturation IL-18 assessed by WES capillary electrophoresis coupled with Ab-based detection in whole cell lysates (WCL) of primary human monocytes stimulated as in C. One experiment is shown with 2 donors.

Appendix Fig. S2 - Platelet depletion induces transcriptional reprogramming in primary human monocytes.

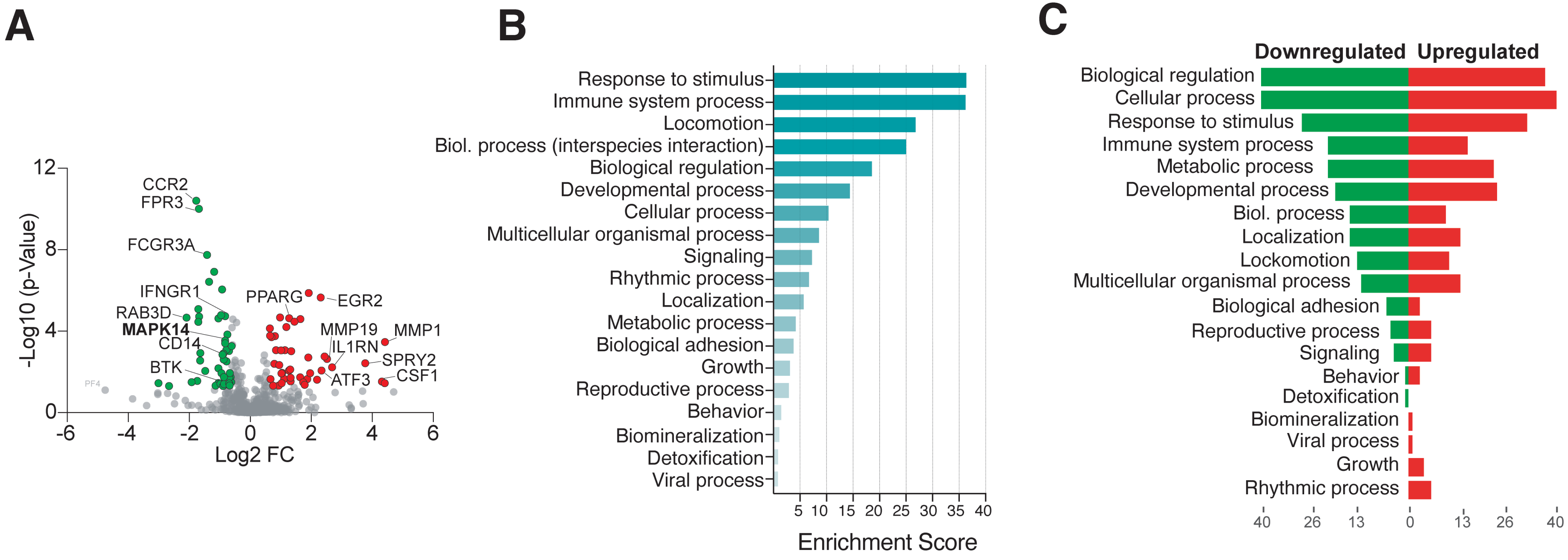

(**A**) Volcano plot displays differentially expressed genes induced by platelet depletion alone comparing unstimulated MoPD and MoStd. Volcano plot shows significantly upregulated (red) and downregulated (green) genes.

(**B**) Gene ontology (GO) analysis displays enriched gene sets categorized in biological processes that were changed upon platelet depletion.

(**C**) Forest plot presents GO analysis showing the proportions of down- (green) and upregulated (red) enriched gene sets in each GO category. Alterations with fold change ≥ 2 and p-value < 0.05 are considered as significant.

Appendix Fig. S3 - Bulk RNASeq analysis of FACS-sorted murine monocytes from platelet-depleted mice.

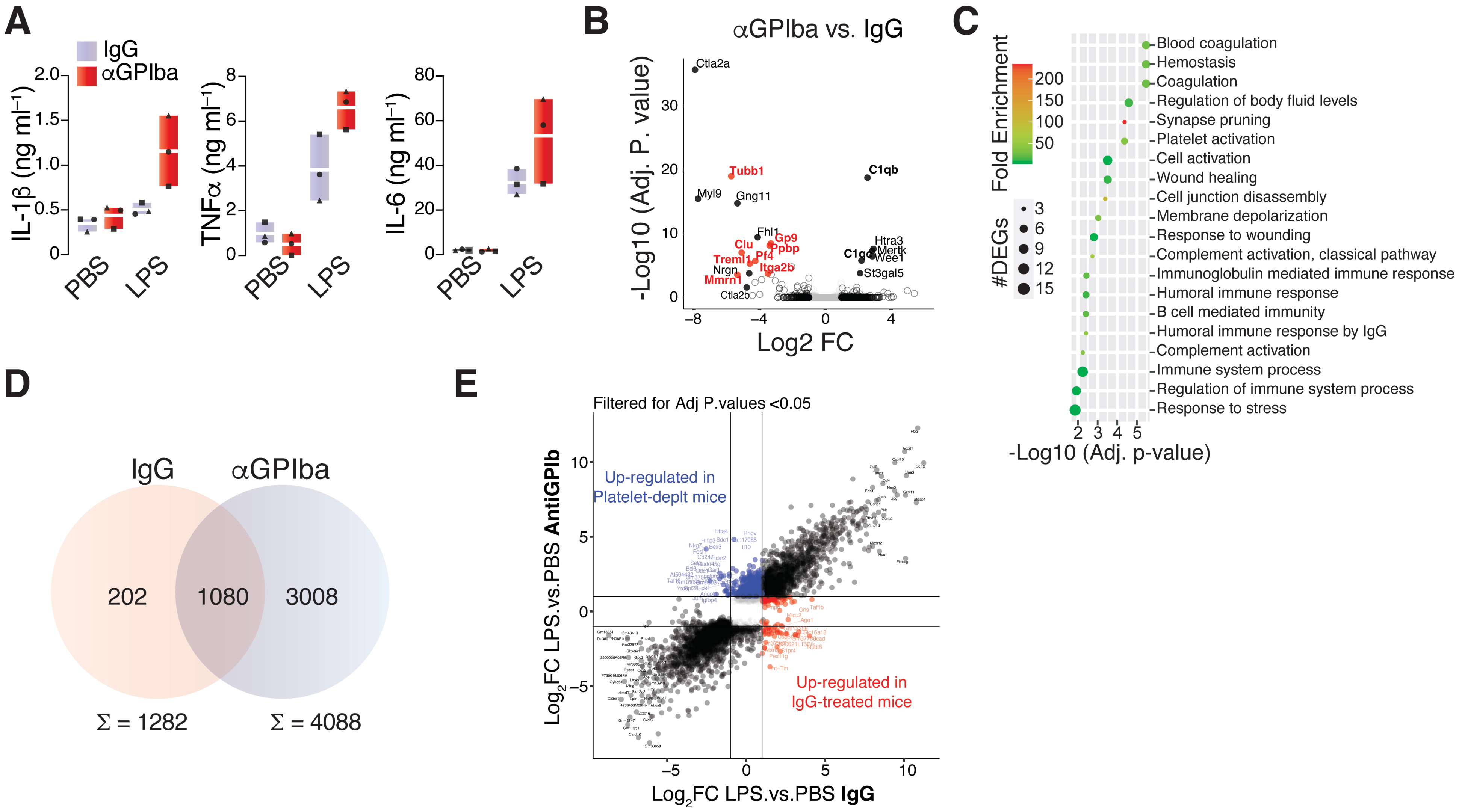

(**A**) Plasma cytokine levels in IgG or anti-GPIbα-treated mice challenged with LPS, or vehicle (PBS).

(**B - C**) Volcano plot showing the log2 fold change (x-axis) and significance (−log10 *p-value; y-axis), and (C) pathway enrichment analysis of the differentially expressed genes (DEGs, adjusted P value < 0.05, Fold Change >=2, <= -2) comparing PBS-challenged anti-GPIba-treated vs. IgG-treated mice. Platelet-specific transcripts are highlighted in red.

(**D**) Venn-diagram of the DEGs in monocytes taken in IgG (1282) vs. anti-GPIba-treated mice (4088).

(**E**) Scatter plot comparing the DEGs (-2 <= Fold Change >= 2, FDR corrected P.value < 0.05) of LPS-induced gene expression in monocytes isolated from IgG-treated (x.axis) vs. anti-GPIbα-treated mice (y.axis).

Appendix Data S4 - S5 - Platelets regulate cytokine secretion in human monocytes in trans and independently of classical platelet-monocyte crosstalk mechanisms.

Evidence highlights that innate immune cells can engulf platelets (Lang et al, 2002; Maugeri et al, 2009; Rolfes et al, 2020; Senzel & Chang, 2013). Our observations did not reveal distinguishable internalization of platelets by human monocytes (Appendix Fig. **S4A**). However, blocking actin polymerization in platelet-depleted monocytes (PdMo) via Cytochalasin D (CytoD) (Appendix Fig. **S4B)** or Latrunculin B (Lat-B) (Appendix Fig. **S4A**) ablated the ability of platelets to restore their faulty cytokine secretion. The potential role of endocytosis in this mechanism was also assessed, but the treatment of PdMo with Dynasore (DS), an inhibitor of dynamin 1/2 endocytosis, did not prevent the platelets' ability to reconstitute the faulty cytokine response of PdMo (Appendix Fig. **S4B**). These data underpins the significance of monocyte's phagocytic machinery for the influence exerted by platelets, albeit without directly visible platelet phagocytosis, supporting a scenario where platelet-derived vesicles (PMPs), although too small for imaging, are internalized by monocytes (Vajen et al, 2015). Supporting this conclusion, platelet released molecules (Plts Sups) efficiently restored cytokine production in LPS-stimulated human PdMo (Appendix Fig. **S4D**).

Lastly, we examined whether ligand-receptor interactions mediate platelet-monocyte crosstalk. A well-documented mechanism of monocyte-platelet or monocyte-PMP binding hinges on the interaction between platelet P-selectin (P-sel or CD62P) and P-selectin glycoprotein ligand-1 (PSGL-1 or CD162) present on monocytes (Frenette et al, 2000; Han et al, 2020). To delineate the importance of the Psel-PSGL-1 axis, we employed KPL-1, an antibody targeting P-sel. Although KPL-1 markedly reduced monocyte-platelet binding (Appendix Fig. **S4E-F**), it neither inhibited pro-inflammatory cytokine production in stimulated StdMo nor impeded platelets in restoring cytokine dysfunction in PdMos (Appendix Fig. **S4H**).

Although both soluble and immobilized P-sel can influence innate immune functions in stimulated monocytes (Weyrich et al, 1995), and platelets can release P-sel in vesicles (Forlow et al, 2000), introducing soluble recombinant human P-sel to PdMo didn't rectify their impaired cytokine secretion, compared to the re-addition of autologous platelets (Appendix Fig. **S5A**). These insights suggest the P-sel-PSGL-1 interaction is not central to revitalizing the faulty cytokine response of PdMo. Despite KPL-1 causing a physical separation between platelets and StdMo (Appendix Fig. **S4E-G**), it did not hinder cytokine secretion, supporting the involvement of vesicles that may still be present.

As the platelet-monocyte crosstalk is additionally facilitated by platelet glycoprotein-Ib (GPIIb) and monocyte CD11b (Carestia et al, 2019; Malehmir et al, 2019), we blocked this ligand-receptor axis with anti-CD11b, or the GPIIb/IIIa inhibitor trihydrochloride (GR-144053) (Appendix Fig. **S5B**). Notably, neither targeting the CD11b/CD18 (MAC-1) complex nor inhibiting GPIIb/IIIa prevented the platelet's ability to rescue the faulty cytokine production in platelet-depleted monocytes (Appendix Fig. **S5B**). Next, we blocked the bulk class of integrins involved in platelet adhesion, and interactions with leukocytes (Bennett et al, 2009; Nieswandt et al, 2000). We used RGD, an arginine-glycine-aspartate tripeptides (RGDs) to mask the binding sites of integrins (Haskel & Abendschein, 1989; Ruoslahti, 1996). We pre-incubated platelets with RGD before their addition to PdMo. Irrespective of RGD, platelets similarly rescued cytokine secretion of PdMo (Appendix Fig. **S5C**), indicating that this class of integrins is not involved in the platelet regulation of monocyte cytokine responses.

We next targeted known proteins expressed and released by platelets. Platelets produce RANTES (CCL5) and SDF1a (CXCL12), which regulate inflammatory functions of monocytes(Alard et al, 2015; Chatterjee et al, 2015). However, in contrast to platelets, the supplementation of PdMo with rhCCL5 or rhCXCL12 did not rescue their faulty cytokine secretion (Appendix Fig. **S5D**). Furthermore, these cytokines were not consistently detected in platelets by our Luminex assays (Fig. **1J**; and **EV1K**).

As demonstrated in our protein kinase assay (Fig. **5**), the activation of the CD40-CD40L axis figured in the top pathways regulated by platelet-monocyte interactions (Fig **5E**). This is consistent with the description of CD40L expression on platelets. CD40L binding to CD40 is a co-stimulatory signal for cytokine secretion in macrophages(Henn et al; Inwald et al, 2003). However, supplementation of PdMo with soluble rhCD40L did not substitute platelets in their ability to rescue the impaired cytokine secretion (Appendix Fig. **S5E**). Furthermore, blockage of the CD40-CD40L axis with anti-CD40L mAb had no influence on the platelet rescue of cytokines in PdMos (Appendix Fig. **S5F-H**). These findings conclusively exclude the CD40-CD40L axis as the mechanism for the interdependency of monocytes on platelets for their cytokine responses.

Platelets contain sialic acids (SAs), which are implicated in aggregation and adhesion to leukocytes. To examine whether platelet SAs influences monocytes-derived cytokine response, we generated desialylated platelets (dsPlts) by Neuraminidase from *Arthrobacter ureafaciens* treatment. dsPlts were equally able to rescue cytokine secretion in PdMo as intact platelets. Consistent with previous reports of hyperactivity in dsPlts(Kullaya et al, 2018), supplementation of PdMo with dsPlts induced higher cytokine levels as intact platelets (Appendix Fig. **S5I**). To further examine the role of SAs in the platelet-monocyte crosstalk, we tested whether platelets crosslink SA-binding Ig-like lectin-7 (Siglec-7) on monocytes to drive a pro-inflammatory state of monocytes(Varchetta et al, 2016). However, neither plate-bound nor soluble anti-Siglec-7 rescued the impaired cytokine secretion in PdMos (Appendix Fig. **S5J**).

Together, these findings indicate that the classical and well-described mechanisms of platelet-monocyte crosstalk (i.e., co-stimulatory molecules, integrins, CD40-CD40L axis, CCL5, CXCL12, and SAs) are not involved in the effects described in our study.

Appendix Fig. S4 - Platelets modulate monocyte cytokine responses in trans.

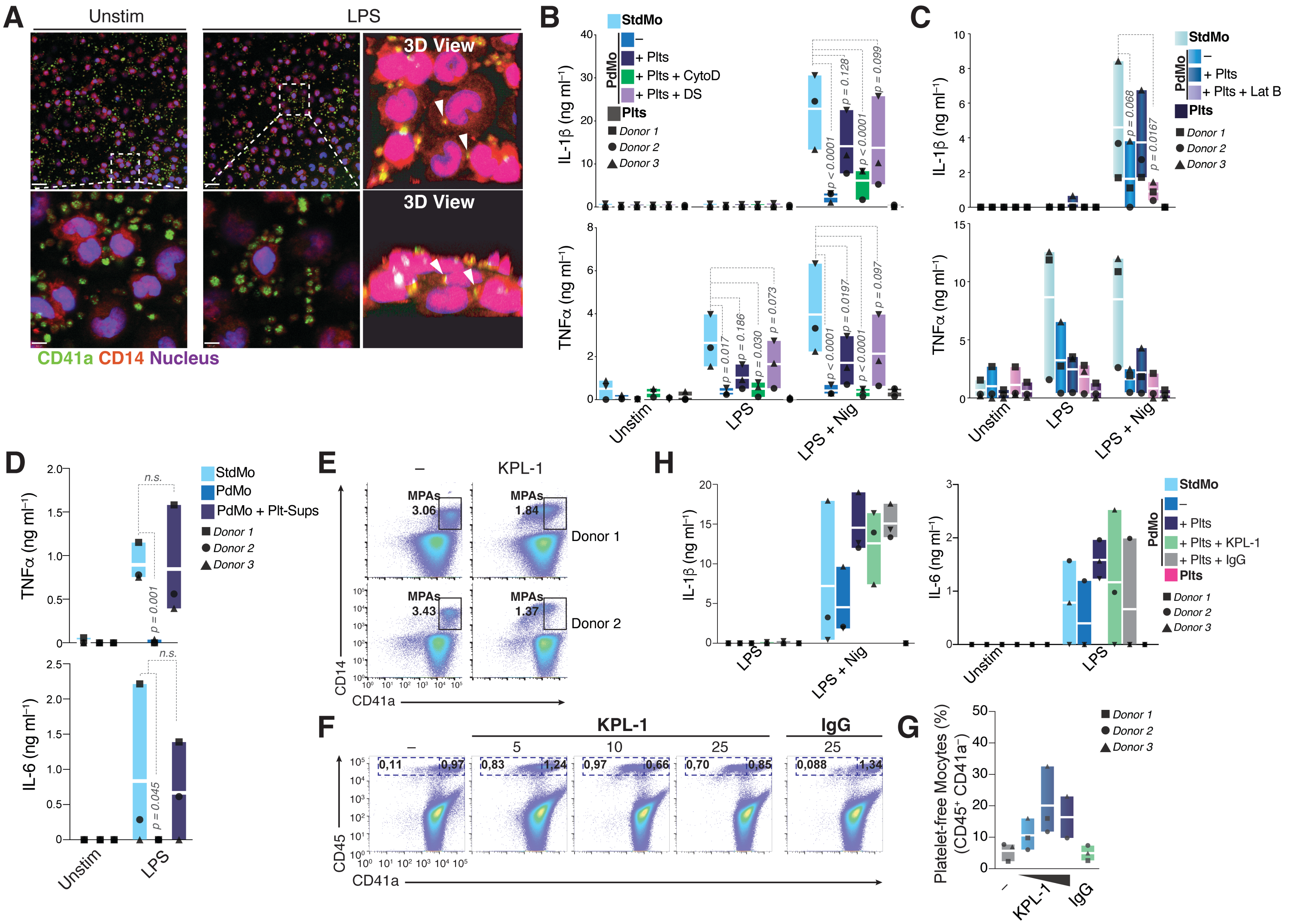

(**A**) Confocal imaging of StdMo and platelets. Cells were stained with CD41a AF488 (platelets: green) and CD14 AF647 (monocytes: red). Nuclei were stained with Hoechst 34580 (blue). Scale: 24 µm (top panel); 4.8 µm (bottom panel). White arrows indicate points of contacts between platelets and monocytes. Images are from one representative of four independent experiments.

(**B**) IL-1β and TNFα levels in CFS of StdMo, PdMo, and PdMo that were supplemented with autologous platelets (PdMo + Plts, 100:1 platelets/ monocytes). Monocytes were pre-treated with Cytochalasin D (CytoD, 50 µM) or Dynasore (DS, 30 µM) before being supplied with Plts. Cells were stimulated with LPS (2 ng ml-1), followed by activation with nigericin (10 µM). N = 3 biological replicates.

(**C**) IL-1β and TNFα levels in CFS of StdMo, PdMo, and PdMo that were supplemented with autologous platelets (PdMo + Plts, 100:1 platelets/monocytes). Monocytes were pre-treated with Lacuntrulin B (Lat B, 2 µM), before being supplied with Plts. Cells were stimulated with LPS (2 ng ml-1), followed by activation with nigericin (10 µM). N = 3 biological replicates.

(**D**) TNFα and IL-6 levels in CFS of LPS stimulated StdMo, PdMo, PdMo, or PdMo reconstituted with platelet releasates (Plt-Sups). N = 3 biological replicates.

(**E**-**G**) Representative flow cytometry assessment and gating strategy (**E-F**) and (**G**) cumulative frequencies of free monocytes (CD45+ CD41a-) in StdMo that were incubated with growing concentrations of KPL-1 (5, 10 or 25 µg ml^{-1}), or IgG (25 µg ml^{-1}). Gates indicate the frequencies of free platelets ($CD45^{-}$ $CD41a^{+}$), MPAs ($CD45^{+}$ $CD41a^{+}$) and platelet-free monocytes ($CD45^{+}$ $CD41a^{-}$). Data is representative of two independent experiments with several donors.

(**H**) IL-1β, TNFα and IL-6 levels released by stimulated StdMo that were treated with increasing concentrations of KPL-1 (5, 10 or 25 µg ml-1), or IgG (25 µg ml-1).

Graphs display floating bars with the max/min values and mean (white bands) combined from multiple experiments. P values were calculated with 2-Way Anova, Tukey's multiple comparison test, and are displayed in the Fig. Each symbol represents one independent experiment or blood donor.

Appendix Fig. S5: Platelet effects are independent of classical cross-talk mechanisms and sialic acids.

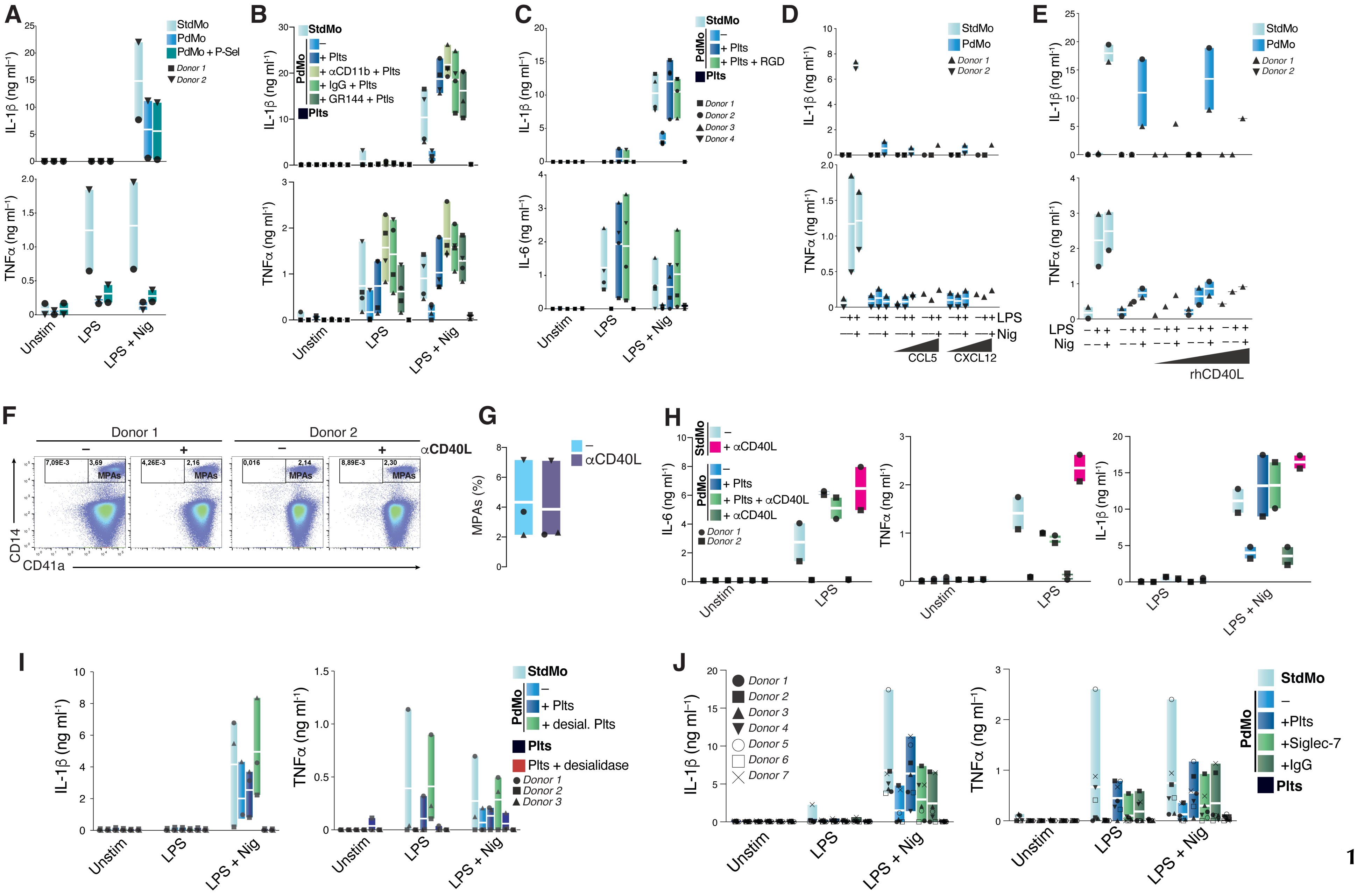

(A) IL-1β and TNFα levels in CFS of StdMo, PdMo, and PdMo that were supplemented with recombinant human CD62P (P-Sel, 40 ng ml–1). Cells were stimulated with LPS (2 ng ml^{-1}), followed by activation with nigericin (10 µM). N = 2 biological replicates.

(B) IL-1β, and TNFα levels in CFS of StdMo, PdMo, PdMo, or PdMo + Plts and stimulated with LPS or LPS + Nig. PdMo were re-added with platelets in the presence of a blocking mAb against CD11b (10 µg·ml-1), or 10µM of the inhibitor of GPIIb/IIIa (GR 144053). N = 4 biological replicates.

(C) IL-1β and IL-6 levels in CFS of StdMo, PdMo, PdMo, or PdMo that were supplemented with untreated (+ Plts) or platelets treated with Arginine-Glycine-Aspartate (RGD) peptides, and stimulated as in **A**. N = 4 biological replicates.

(D) IL-1β and TNFα levels in CFS of StdMo, PdMo, PdMo, or PdMo that were supplemented with platelets (+ Plts) or rhCXCL12 (2 - 4 ng ml^{-1}), or rhCCL5 (300 - 600 µg ml^{-1}), and stimulated as in **A**. N = 2 biological replicates.

(E) IL-1β, and TNFα levels in CFS of StdMo, PdMo, PdMo, or PdMo + Plts and stimulated with LPS or LPS + Nig. PdMo were supplemented with platelets (+ Plts) or rhCD40L (10, 50, or 100 ng ml-1). N = 2 biological replicates.

(F - G) Representative flow cytometry assessment with gating strategy, and quantification (C) of StdMo that were incubated with a monoclonal Ab against CD40L. Gates indicate the frequencies of platelet-free monocytes (CD45+ CD41a–) and MPAs (CD14+ CD41a+). N = 2 biological replicates.

(H) IL-6, TNFα and IL-1β levels in CFS of StdMo, PdMo, PdMo, or PdMo that were supplemented with untreated (+ Plts) or platelets treated with 50µM of Arginine-Glycine-Aspartate (RGD) peptides, and stimulated as in A. As control, anti-CD40L mAb were added directly to StdMos. N = 2 biological replicates.

(I) IL-1β and TNFα levels in CFS of StdMo, PdMo, PdMo, or PdMo that were supplemented with intact (+ Plts), or desialylated platelets (desial. Ptls), and stimulated as in A. N = 3 biological replicates.

(J) IL-1β and TNFα levels in CFS of StdMo, PdMo, PdMo, or PdMo that were supplemented with untreated platelets or platelet-bound to anti-siglec7 (10 µg·ml-1), and stimulated as in A. N = 7 biological replicates.

All graphs show floating bars display max/min values with indication to the mean (white bands). Each symbol represents one donor, or independent experiment. P values were calculated with 2-Way Anova, Tukey's multiple comparison test, and are displayed in the figures.

Appendix Fig S6 - Analysis of Mass speck proteomics combined with Stable isotope labeling with amino acids in cell culture (SILAC)

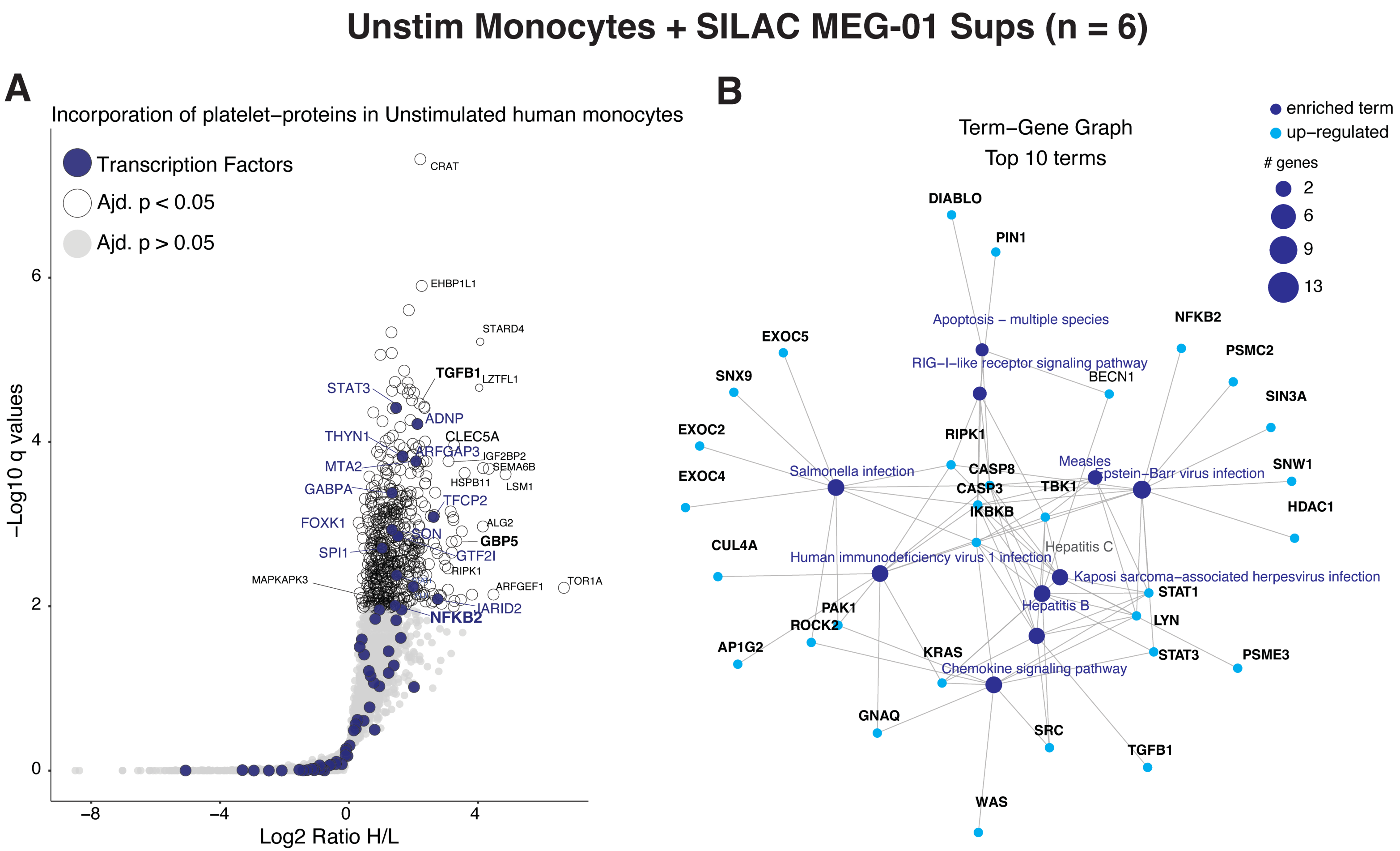

(A) Volcano plots of proteins with heavy-AAs detected in PdMo exposed to cell-free supernatants of MEG-01 cells through Mass speck proteomics combined with Stable isotope labeling with amino acids in cell culture (SILAC). Unstimulated conditions are shown (n = 6 biological replicates). LPS-stimulated conditions are shown in Fig 6.

(B) Pathway analysis of the proteins with heavy-AAs detected in PdMo exposed to cell-free supernatants of MEG-01 cells as in A.

Appendix Fig. S7 - The IKK inhibitor (BAY11-7082) prevents platelet activation.

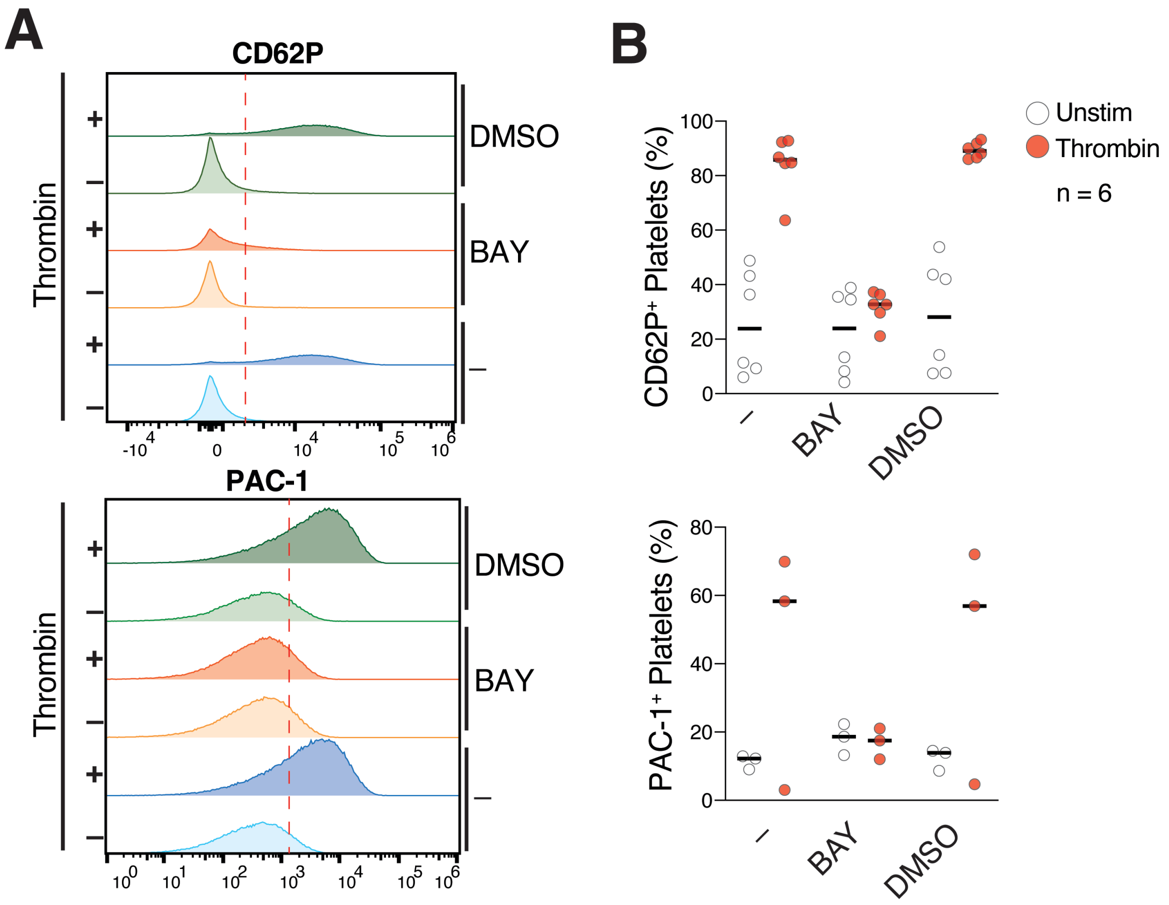

(**A - B**) Flow cytometry analysis and (**B**) quantification of CD62P and PAC-1 expression on purified human platelets. Platelets were left untreated (–), or treated with BAY11-7082 (50 µM), DMSO (50 µM) for 20 min, before being stimulated with Thrombin (1 U ml^{-1}) for 30 min. Left: Representative FACS histogram for CD62P expression. (**B**) Dotplot displays the percentage of CD62P+ platelets in n=6 different donors with the indication to the mean.

Appendix Fig. S8 - Fractionation of platelet releasates and isolation of platelet vesicles.

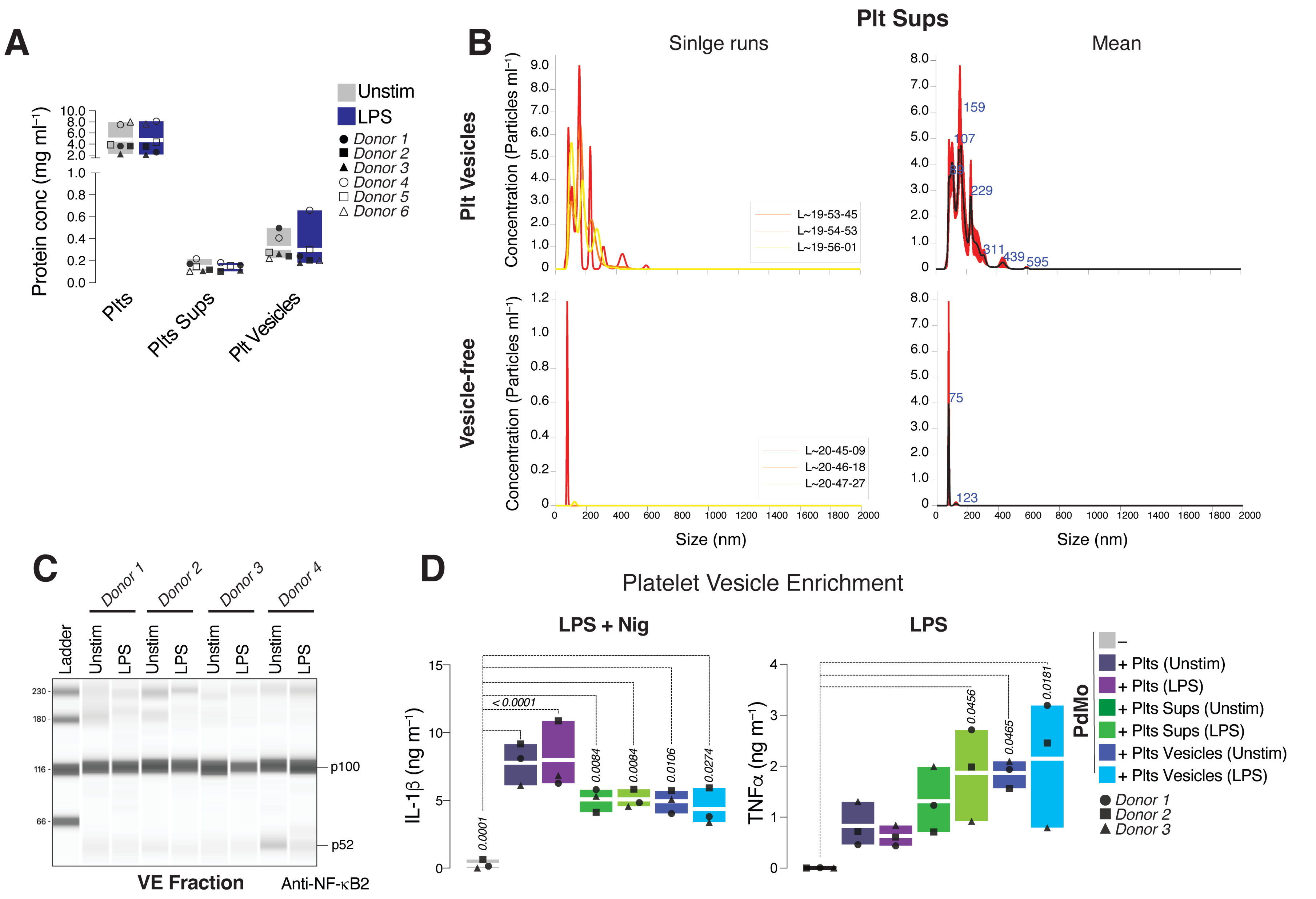

(**A**) Protein concentrations of platelet lysates, platelet releasates (Plt Sups), or platelet vesicles measured by Pierce™ BCA Protein Assay Kit (Thermo Fisher Scientific). Conditions included treatment with LPS (2 ng ml^{-1}) for 3 hours or no treatment. Each symbol represents one donor.

(B) Vesicle analysis of the different platelet supernatants using NanoSight3000. Each sample was diluted 1:10 with PBS and 1 ml total volume was used and recorded three times for 60 seconds before analysis. On the left are three individual runs of the same condition (marked by different colors: yellow, orange, and red). On the right is the calculated mean. The Y-axis represents the concentration of particles detected per milliliter, and the X-axis shows the size (nm) of the vesicles/particles. Each graph represents one independent experiment or donor.

(**C**) WES-capillary electrophoresis and immunodetection of NF-κB2 from vesicle-enriched platelets from 4 healthy subjects. Platelets were left untreated (Unstim), or stimulated or restimulated with 2 ng/ml LPS for 3 hours before the assessment.

(**D**) IL-1β and TNFα levels in the cell-free supernatant (CFS) of PdMo and PdMo supplemented with autologous platelets, platelet releasates (Plt Sups), or concentrated platelet vesicles. Platelets, Plt Sups, or vesicles were pre-treated with LPS (2 ng ml^{-1}) for 3 hours before addition to PdMo. Cells were stimulated with LPS (2 ng ml^{-1}) followed by activation with nigericin (10 μM). Graphs with floating bars depict maximum/minimum values relative to the mean (white bands). P values were calculated using 1-way ANOVA, Tukey's multiple comparison test, and are indicated in the figure. Each symbol represents one donor.

Appendix References

Alard J-E, Ortega-Gomez A, Wichapong K, Bongiovanni D, Horckmans M, Megens RTA, Leoni G, Ferraro B, Rossaint J, Paulin N *et al* (2015) Recruitment of classical monocytes can be inhibited by disturbing heteromers of neutrophil HNP1 and platelet CCL5. *Sci Transl Med* 7: 317ra196-317ra196

Bennett JS, Berger BW, Billings PC (2009) The structure and function of platelet integrins. *J Thromb Haemost* 7 Suppl 1: 200-205

Carestia A, Mena HA, Olexen CM, Ortiz Wilczyñski JM, Negrotto S, Errasti AE, Gómez RM, Jenne CN, Carrera Silva EA, Schattner M (2019) Platelets Promote Macrophage Polarization toward Pro-inflammatory Phenotype and Increase Survival of Septic Mice. *CellReports* 28: 896-908.e895

Chatterjee M, von Ungern-Sternberg SNI, Seizer P, Schlegel F, Büttcher M, Sindhu NA, Müller S, Mack A, Gawaz M (2015) Platelet-derived CXCL12 regulates monocyte function, survival, differentiation into macrophages and foam cells through differential involvement of CXCR4–CXCR7. *Cell Death and Disease* 6: e1989-e1989

Forlow SB, McEver RP, Nollert MU (2000) Leukocyte-leukocyte interactions mediated by platelet microparticles under flow. *Blood* 95: 1317-1323

Frenette PS, Denis CV, Weiss L, Jurk K, Subbarao S, Kehrel B, Hartwig JH, Vestweber D, Wagner DD (2000) P-Selectin glycoprotein ligand 1 (PSGL-1) is expressed on platelets and can mediate platelet-endothelial interactions in vivo. *J Exp Med* 191: 1413-1422

Han P, Hanlon D, Arshad N, Lee JS, Tatsuno K, Robinson E, Filler R, Sobolev O, Cote C, Rivera-Molina F *et al* (2020) Platelet P-selectin initiates cross-presentation and dendritic cell differentiation in blood monocytes. *Sci Adv* 6: eaaz1580

Haskel EJ, Abendschein DR (1989) Deaggregation of human platelets in vitro by an RGD analog antagonist of platelet glycoprotein IIb/IIIa receptors. *Thromb Res* 56: 687-695

Henn V, Slupsky JR, Grafe M, Anagnostopoulos I, Forster R, Muller-Berghaus G, Kroczek RA (1998) CD40 ligand on activated platelets triggers an inflammatory reaction of endothelial cells. *Nature* 391: 591-594

Henn V, Slupsky JR, Gräfe M, Nature IA, 1998 CD40 ligand on activated platelets triggers an inflammatory reaction of endothelial cells. *naturecom*

Inwald DP, McDowall A, Peters MJ, Callard RE, Klein NJ (2003) CD40 is constitutively expressed on platelets and provides a novel mechanism for platelet activation. *Circ Res* 92: 1041-1048

Kullaya V, de Jonge MI, Langereis JD, van der Gaast-de Jongh CE, Bull C, Adema GJ, Lefeber D, Cremers AJ, Mmbaga BT, de Groot PG *et al* (2018) Desialylation of Platelets by Pneumococcal Neuraminidase A Induces ADP-Dependent Platelet Hyperreactivity. *Infect Immun* 86

Lang D, Dohle F, Terstesse M, Bangen P, August C, Pauels HG, Heidenreich S (2002) Down-regulation of monocyte apoptosis by phagocytosis of platelets: involvement of a caspase-9, caspase-3, and heat shock protein 70-dependent pathway. *J Immunol* 168: 6152-6158

Malehmir M, Pfister D, Gallage S, Szydlowska M, Inverso D, Kotsiliti E, Leone V, Peiseler M, Surewaard BGJ, Rath D *et al* (2019) Platelet GPIbalpha is a mediator and potential interventional target for NASH and subsequent liver cancer. *Nat Med* 25: 641-655

Maugeri N, Rovere-Querini P, Evangelista V, Covino C, Capobianco A, Bertilaccio MT, Piccoli A, Totani L, Cianflone D, Maseri A, Manfredi AA (2009) Neutrophils phagocytose activated platelets in vivo: a phosphatidylserine, P-selectin, and {beta}2 integrin-dependent cell clearance program. *Blood* 113: 5254-5265

Nieswandt B, Bergmeier W, Rackebrandt K, Gessner JE, Zirngibl H (2000) Identification of critical antigen-specific mechanisms in the development of immune thrombocytopenic purpura in mice. *Blood* 96: 2520-2527

Rolfes V, Ribeiro LS, Hawwari I, Bottcher L, Rosero N, Maasewerd S, Santos MLS, Prochnicki T, Silva CMS, Wanderley CWS *et al* (2020) Platelets Fuel the Inflammasome Activation of Innate Immune Cells. *Cell Rep* 31: 107615

Ruoslahti E (1996) RGD and other recognition sequences for integrins. *Annu Rev Cell Dev Biol* 12: 697-715

Senzel L, Chang C (2013) Platelet phagocytosis by neutrophils. *Blood* 122: 1543

Vajen T, Mause SF, Koenen RR (2015) Microvesicles from platelets: novel drivers of vascular inflammation. *Thromb Haemost* 114: 228-236

Varchetta S, Mele D, Lombardi A, Oliviero B, Mantovani S, Tinelli C, Spreafico M, Prati D, Ludovisi S, Ferraioli G *et al* (2016) Lack of Siglec-7 expression identifies a dysfunctional natural killer cell subset associated with liver inflammation and fibrosis in chronic HCV infection. *Gut* 65: 1998-2006

Weyrich AS, McIntyre TM, McEver RP, Prescott SM, Zimmerman GA (1995) Monocyte tethering by P-selectin regulates monocyte chemotactic protein-1 and tumor necrosis factor-alpha secretion. Signal integration and NF-kappa B translocation. *J Clin Invest* 95: 2297-2303
