# Supplementary material for: Platelet transcription factors license the pro-inflammatory cytokine response of human monocytes
Source: EMBO Mol Med. 2024 Jul 8;16(8):6. doi: 10.1038/s44321-024-00093-3 (PMC11319489; doi:10.1038/s44321-024-00093-3)
Supplement: Supplementary file 3 — Expanded View Figures [file 44321_2024_93_MOESM3_ESM.pdf]

Expanded View Figures

Figure EV1. Platelets are critical checkpoints for the cytokine production of primary human monocytes.

(**A**) Representative flow cytometry analysis of human PBMCs, and isolated untouched (StdMo), or platelet-depleted (PdMo) primary $CD14^+$ monocytes stained with CD14 (monocyte marker) and CD41a (platelet marker), or corresponding isotype controls. Gating strategy to identify platelet-free monocytes ($CD14^+$ $CD41a^-$, Monos), monocyte-platelet aggregates ($CD14^+$ $CD41a^+$, MPAs), and free platelets ($CD14^-$ $CD41a^+$, Plts). Plots on the right shows immunophenotyping of PBMCs, StdMo and PdMo based on the surface expression of CD14 and CD16. Data is from one representative of several independent experiments. (**B**) Comparative quantification of Monos, MPAs and Plts populations based on their frequency determined by flow cytometry in monocyte isolations ($n = 50$) as shown in A. Error bars show the SD of each group (Monos, MPAs and Plts). (**C**) Quantification of platelet and monocyte counts through a CASY cell counter and analyzer. (**D**) Confocal imaging of StdMo showing $CD14^+$ monocytes (blue) and $CD61^+$ platelets (red), and the formation of MPAs. Scale bars: 10 μm. (**E**) IL-1β and IL-6 concentrations in cell-free supernatants (CFS, left) or whole cell lysates (WCL, right) from untouched (StdMo), platelet-depleted (PdMo), or PdMo that were supplemented with autologous platelets (100:1 platelet:monocyte ratio). Cytokine levels secreted by platelets alone (Plts) were measured as control. Cells were stimulated with LPS (2 ng ml^{-1} for 3 h) followed by activation with nigericin (10 μM for 1.5 h, for IL-1β), or directly with LPS (2 ng ml^{-1} for 4.5 h, for IL-6). Floating bars display the max/min values with indications of the mean (white bands). Each symbol represents one independent experiment/blood donor. (**F–H**) Flow Cytometry assessment of surface expression of CD14 and TLR4 in StdMo, and PdMo, showing (**F**) gating strategy, (**G**) kinetics and (**H**) comparative quantification of CD14 and TLR4 expression over time (0.5, 1, 2, or 3 h) upon LPS (2 ng ml^{-1}) stimulation. Data display the percentage of $TLR4^+$ or $CD14^+$ StdMo or PdMo in each group. Each symbol represents one of $n = 5$ different donors. (**I**) Cell viability assessed every 15 min in StdMo, PdMo, PdMo + Plts, and Plts treated as in (**E**). (**J**) IL-1β concentrations in CFS of LPS-primed StdMo, PdMo, or PdMo + Ptls that were stimulated with Pam3CSK4 (1 μg ml^{-1}), Resiquimod R848 (10 μM), or LPS (2 ng ml^{-1}) for 4.5 h and left untreated, or further activated with nigericin (10 μM, for 90 min). Floating bars display the max/min values with indications of the mean (white bands). Each symbol represents one independent experiment/blood donor. *P* values are from ANOVA multiple comparison test. (**K**) Radar plots displaying all 45 cytokines, chemokines and growth factors measured by Cytokine Luminex in the CFS of StdMo, PdMo, or PdMo + Ptls and platelets stimulated with Pam3CSK4 (1 μg ml^{-1}) or Resiquimod R848 (10 μM). Protein concentrations are represented by the spread from inner (0 ng ml^{-1}) to outer circles (> 15 ng ml^{-1}). Colours represent stimuli (Unstim, dark gray; Pam3CSK4, blue; and R848, red). Each symbol represents one independent experiment/blood donor ($n = 4$ donors).

© The Author(s)

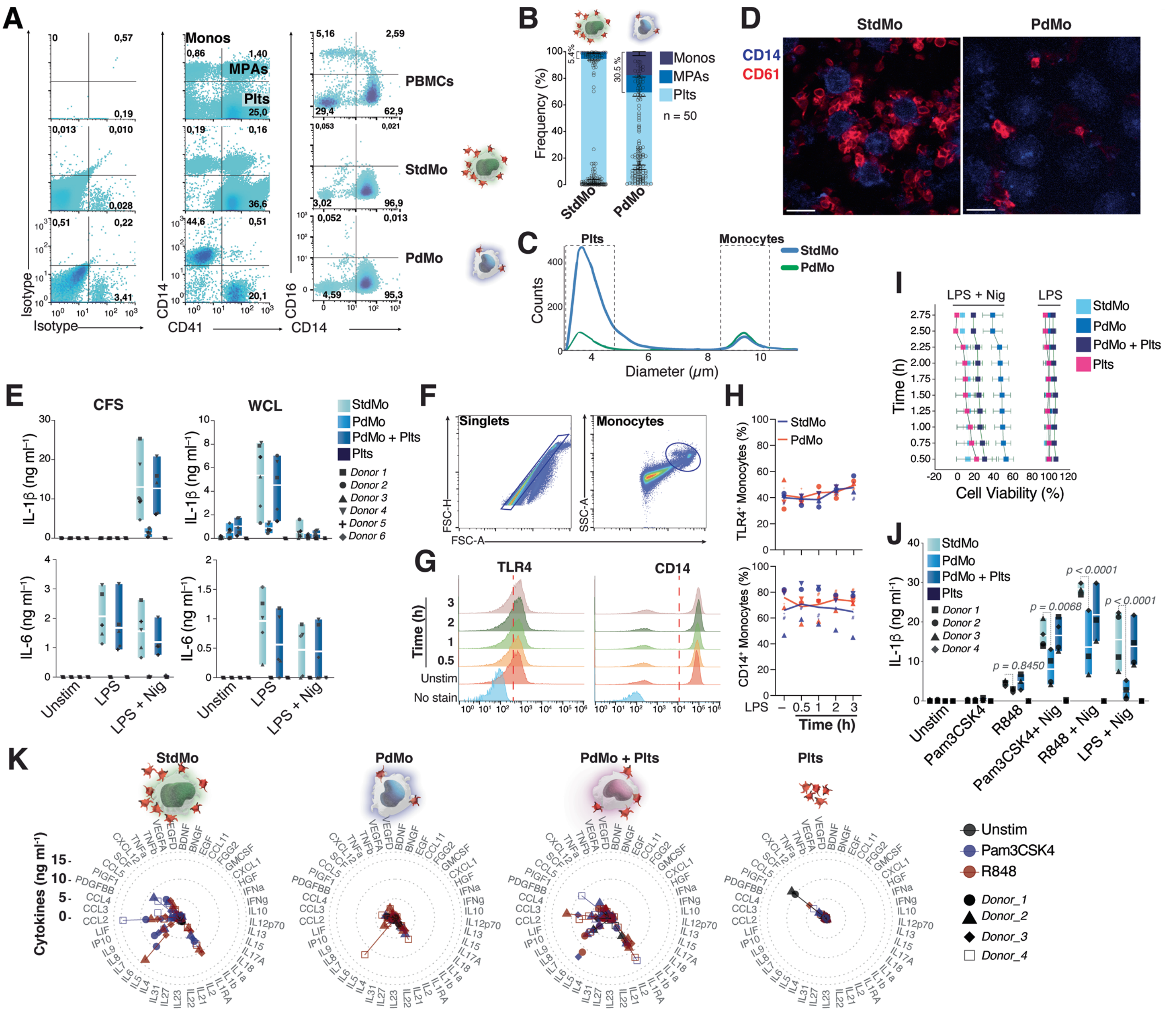
A
Monos
MPAs
Plts
PBMCs
StdMo
PdMo
Isotype
CD14
CD41
CD16
B
Frequency (%)
Monos
MPAs
Plts
n = 50
5.4%
30.5 %
StdMo
PdMo
C
Plts
Monocytes
StdMo
PdMo
Counts
Diameter (μm)
D
StdMo
PdMo
CD14
CD61
E
CFS
WCL
IL-1β (ng ml⁻¹)
IL-6 (ng ml⁻¹)
StdMo
PdMo
PdMo + Plts
Plts
Donor 1
Donor 2
Donor 3
Donor 4
Donor 5
Donor 6
Unstim
LPS
LPS + Nig
F
Singlets
Monocytes
FSC-H
FSC-A
SSC-A
G
TLR4
CD14
Time (h)
Unstim
No stain
H
StdMo
PdMo
TLR4⁺ Monocytes (%)
CD14⁺ Monocytes (%)
LPS
Time (h)
I
LPS + Nig
LPS
StdMo
PdMo
PdMo + Plts
Plts
Time (h)
Cell Viability (%)
J
StdMo
PdMo
PdMo + Plts
Plts
Donor 1
Donor 2
Donor 3
Donor 4
IL-1β (ng ml⁻¹)
p = 0.8450
p = 0.0068
p < 0.0001
p < 0.0001
Unstim
Pam3CSK4
R848
Pam3CSK4+ Nig
R848 + Nig
LPS + Nig
K
StdMo
PdMo
PdMo + Plts
Plts
Cytokines (ng ml⁻¹)
Unstim
Pam3CSK4
R848
Donor_1
Donor_2
Donor_3
Donor_4

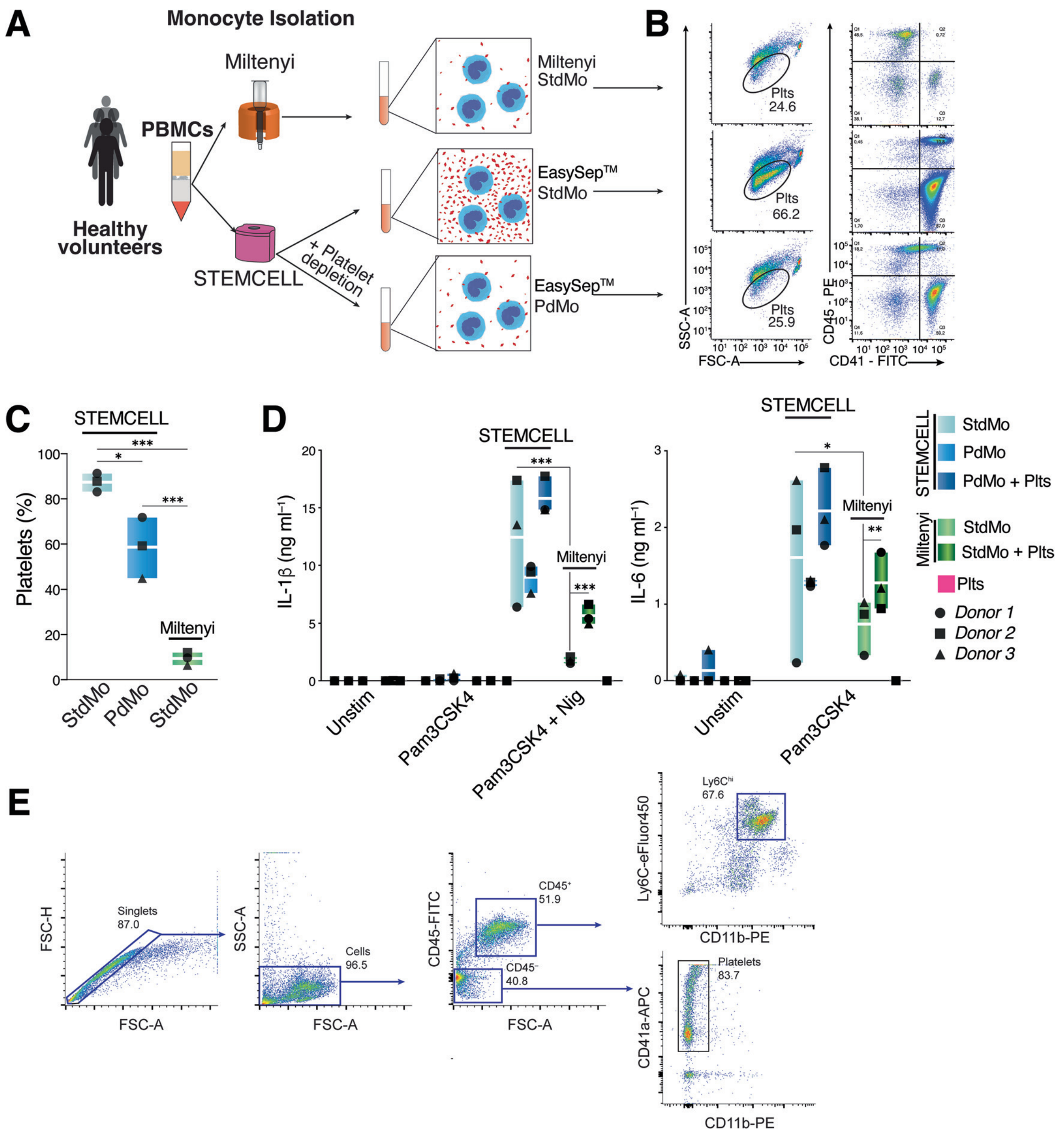

Figure EV2. Impact of platelet removal from primary human monocytes.

(**A**) Schematic presentation of the immune-magnetic isolation of primary human monocytes from peripheral blood comparing the Miltenyi vs. the EasySep™ monocyte isolation kits. The EasySep™ kit was further supplemented with (PdMo) or without (StdMo) a platelet-depletion cocktail. (**B**) Representative flow cytometry analysis of the human primary monocyte populations isolated as in (**A**). Gating shows the populations of platelet-free monocytes (CD14^{+} CD41a^{-}) and platelets (CD41a^{+}), or corresponding isotype controls. Data is from one representative of 3 independent experiments. (**C**) Frequencies of free platelets in populations of monocytes isolated as in (**A**), comparing StdMo (light blue bars), PdMo (blue bars) isolated with the EasySep™ kit, or StdMo isolated with the Miltenyi kit (green bars). Floating bars display the max/min values with indications of the mean (white bands). Each symbol represents one independent experiment/blood donor ($n = 3$). Each symbol represents one independent experiment/blood donor ($n = 3$). *P* values are from two-way ANOVA with Tukey's multiple comparison test with 95% confidence interval, and are indicated as * (<0.05), ** (<0.01), and *** (<0.001). (**D**) Concentrations of IL-1β, and IL-6 released by untouched (StdMo), platelet-depleted (PdMo), or PdMo that were supplemented with autologous platelets (PdMo + Plts, 100:1 platelet:monocyte ratio), using the EasySep™ kit (blue bars), or untouched isolated with the Miltenyi kit cultured alone (StdMo, green bars) or co-cultured with platelets (StdMo + Pts). Cytokine levels secreted by platelets alone (Plts) were measured as control. Cells were stimulated with LPS (2 ng ml^{-1} for 4.5 h, for IL-6) or with LPS (3 h) followed by activation with nigericin (10 μM for 1.5 h, for IL-1β). Floating bars display the max/min values with indications of the mean (white bands). Each symbol represents one independent experiment/blood donor ($n = 3$). *P* values are from two-way ANOVA with Tukey's multiple comparison test with 95% confidence interval, and are indicated as * (<0.05), ** (<0.01), and *** (<0.001). (**E**) Representative Flow Cytometry and gating strategy to assess the purity of murine monocytes isolated from mouse blood. Cells were gated based on surface expression of CD45, Ly6C, and CD41a. Images are from one representative of independent experiments with $n = 4$ mice.

 © The Author(s)

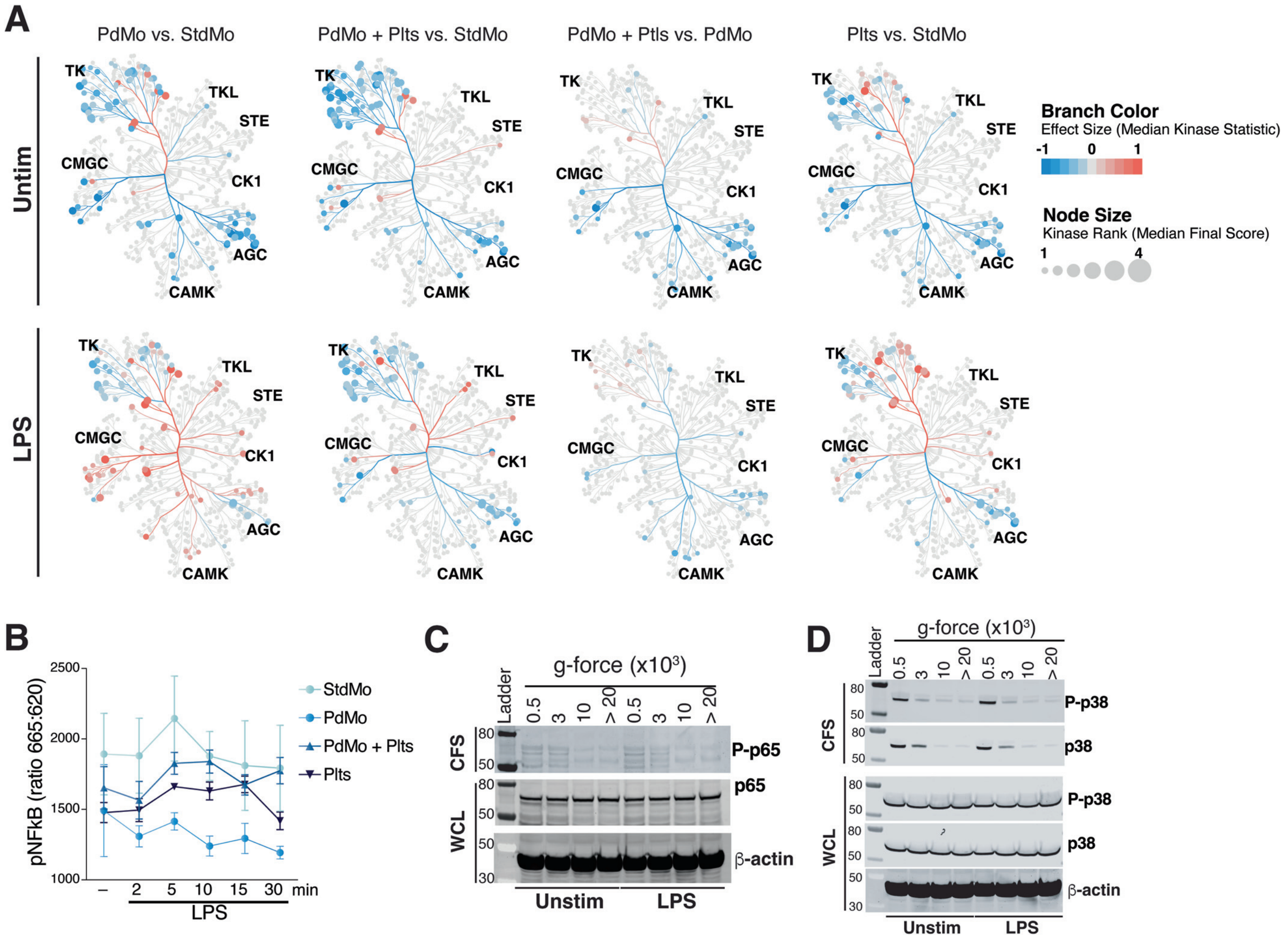

Figure EV3. Intrinsic kinase activity in platelets and their effects on human monocytes.

(**A**) Coral trees displaying the activity of Protein Tyrosine and Serine/Threonine kinases in unstimulated (Unstim) or LPS-treated (LPS; 2 ng ml^{-1}) primary human monocytes comparing the effects of platelet-depletion/supplementation, measured with a PamStation12 (PamGene). (**B**) NF-κB activity assay in CFS of unstimulated (Unstim), or LPS-stimulated (2 ng ml^{-1}, for the indicated times) primary human monocytes (StdMo), PdMo, PdMo + Plts, and Plts alone. Cells were lysed/incubated with lysis buffer, supplied Cisbio, and used to assess phosphorylated NFkB by HTRF (Cisbio). Error bars display the SD ($n = 3$). (**C**, **D**) Immunoblot of RelA (p65 and p-p65) (**C**) or MAPK (p-38 and P-p38) (**D**) in resting (Unstim) or LPS-activated (LPS; 2 ng ml^{-1}) human platelets. Platelets were submitted to centrifugation at 500, 3000, 10,000, or 20,000 × *g* and the levels of proteins were assessed in the pellets (WCL) or supernatants (CSF) after centrifugation. Results are representative of two independent experiments.

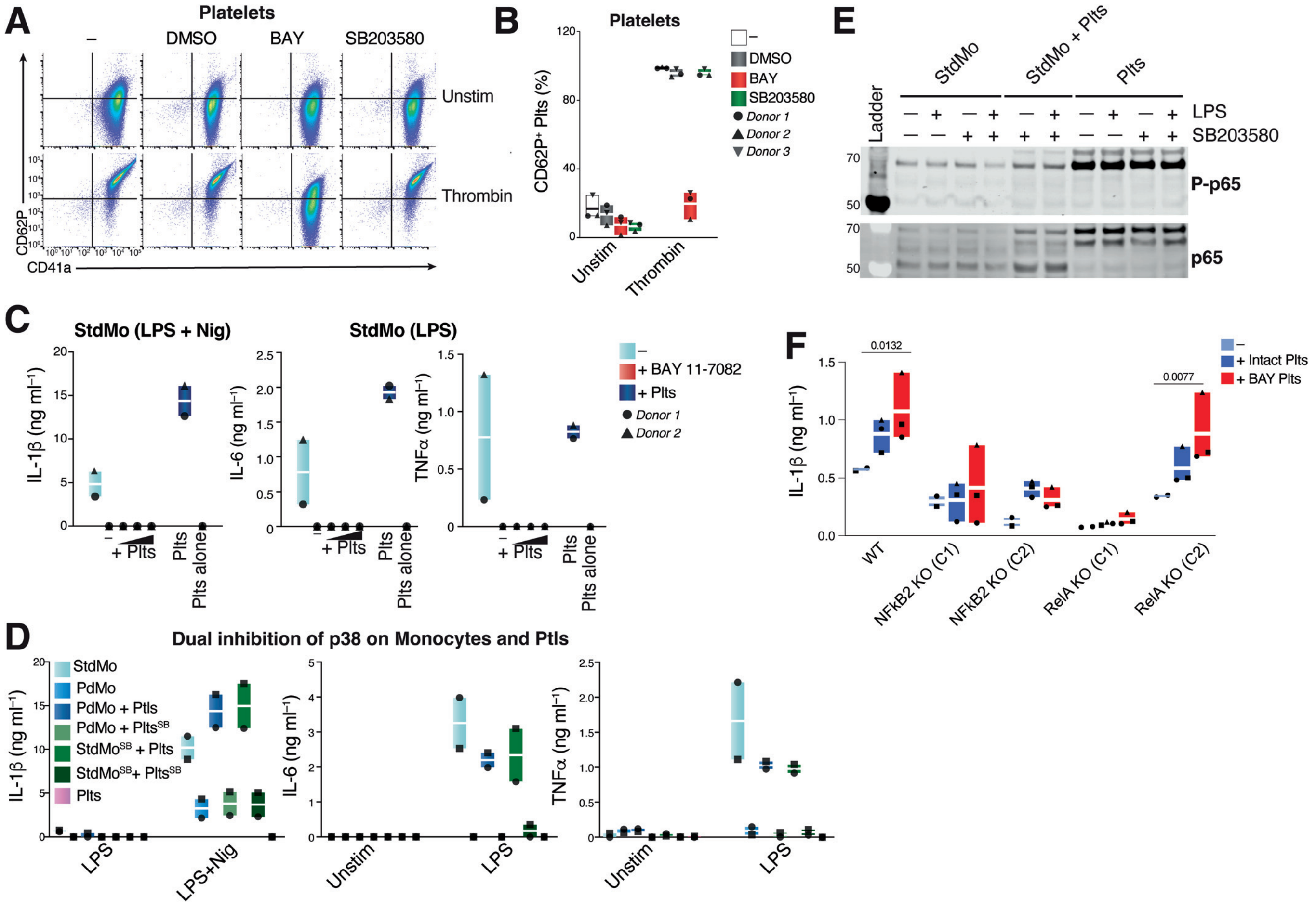

Figure EV4. Dual p38 and NF-kB inhibition on platelets and monocytes.

(**A**, **B**) Representative flow cytometry assessment and (**B**) quantification (%, $n = 3$) of CD41a and P-selectin (CD62P) expression on human platelets that were pre-treated with BAY (50 μM), or SB203580 (20 μM) 20 min before being activated with Thrombin (1 U ml^{-1}) for 30 min. (**C**) IL-1β, TNFα, and IL-6 concentrations in the CFS of StdMo that were treated with BAY before been added with increasing ratios of freshly isolated platelets (1:5, 1:50, and 1:100; StdMo:Plts). Cells were stimulated with LPS or LPS and nigericin (LPS + Nig). Floating bars display max/min values with indication to the mean (white bands). Each symbol represents one donor. (**D**) Concentrations of IL-1β, IL-6, and TNFα in CFS of StdMo, PdMo, or PdMo that were supplemented with platelets pre-treated with 20 μM SB203580 (Plts + SB), or left untreated (+ Plts), or in StdMo pre-treated with SB203580 (StdMo + SB) that were supplemented with intact platelets (StdMo + SB + Plts) or with SB203580-treated platelets (StdMo + SB + Plts + SB). Co-cultures were stimulated with LPS (2 ng ml^{-1}) followed by nigericin stimulation (10 μM) ($n = 2$). (**E**) Immunoblotting for phospho-p65 and total p65 (RelA) on unstimulated and LPS-stimulated StdMo primary human monocytes treated with 20 μM of SB203580. (**F**) IL-1β concentrations in CFS of stimulated $RelA^{-/-}$, $NF\kappa B2^{-/-}$, or the parental WT THP-1 monocytes supplemented with platelets pre-treated with BAY 11-7082 (50 μM, 30 min) before addition to the clones. Cells were stimulated with Pam3CysK4 (1 μg ml^{-1}) or Pam3CysK4 and nigericin (10 μM) (Pam3 + Nig). All Graphs with floating bars depict maximum/minimum values relative to the mean (white bands). *P* values were calculated using two-way ANOVA, Tukey's multiple comparison test, and are indicated in the figure. Each symbol represents one independent experiment or blood donor ($n = 3$).

 © The Author(s)

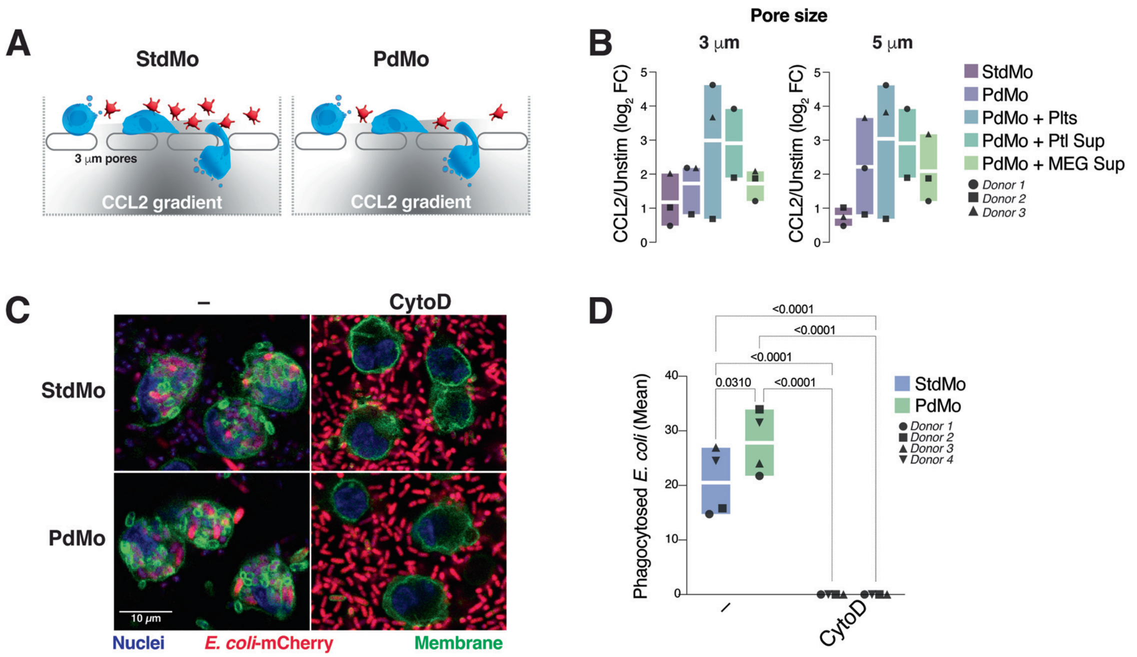

Figure EV5. Effects of platelets on monocyte trans-migration and phagocytosis.

(**A**, **B**) Primary human monocytes (StdMo, or PdMo) were supplemented with platelets, platelet (Plt Sups) or Mk releasates (MK Sups). Cells were then seeded on the upper chamber of a trans well plate with either 3 µm or 5 µm pore sizes and incubated with CCL2 (40 ng ml^{-1}) or left untreated for 4 h. Monocyte trans-migration was measured by confocal imaging and quantification of cells that migrated to the bottom wells. 16 pictures per well were taken and the nuclei (stained with DRAQ5) were counted via Cell Profiler. Log2 fold change was calculated and the CCL2 conditions were normalized to their respective unstimulated condition ($n = 3$). (**C**, **D**) Confocal Imaging (**C**) and quantification (**D**) of StdMo and PdMo exposed to an mCherry fluorescent *E. coli* strain (1:25 MOI, for 2 h). Monocytes were either left untreated (—) or pre-treaded with the phagocytosis inhibitor cycochalasin D (CytoD) and exposed to 100:1 MOI of *E. coli*. Cells were stained with DRAQ5 (blue, nuclei) and WGA-AF488 (green, membranes). Images were acquired by confocal and automated widefield microscopy. Images (four per condition) were analyzed by counting the *E. coli* inside five monocytes per image, by two independent experimenters. Mean numbers of *E. coli* per cell were plotted. Graphs with floating bars depict maximum/minimum values relative to the mean (white bands). *P* values were calculated using two-way ANOVA, Tukey's multiple comparison test. Each symbol represents one donor ($n = 4$).
